# Hemispheric Asymmetries in Price Estimation: Do Brain Hemispheres Attribute Different Monetary Values?

**DOI:** 10.3389/fpsyg.2017.02042

**Published:** 2017-11-22

**Authors:** Felice Giuliani, Anita D’Anselmo, Luca Tommasi, Alfredo Brancucci, Davide Pietroni

**Affiliations:** ^1^Department of Neurosciences, Imaging and Clinical Sciences, Università degli Studi “G. d’Annunzio” Chieti – Pescara, Chieti, Italy; ^2^Department of Psychological Science, Health and Territory, Università degli Studi “G. d’Annunzio” Chieti – Pescara, Chieti, Italy

**Keywords:** hemispheric asymmetries, price estimation, weight estimation, visual half-field stimulation, valence hypothesis

## Abstract

The Spatial Numerical Association of Response Codes (SNARC) effect has been associated with a wide range of magnitude processing. This effect is due to an implicit relationship between numbers and horizontal space, according to which weaker magnitudes and smaller numbers are represented on the left, whereas stronger magnitudes and larger numbers are represented on the right. However, for some particular type of magnitudes such as price, judgments may be also influenced by perceived quality and thus involving valence attribution biases driven by brain asymmetries. In the present study, a lateralized tachistoscopic presentation was used in a price estimation task, using a weight estimation task as a control, to assess differences in asymmetries between these two attributes. Results show a side bias in the former condition but not in the latter, thus indicating that other non-numerical mechanisms are involved in price estimation. Specifically, prices were estimated lower in the left visual field than in the right visual field. The proposed explanation is that price appraisal might involve a valence attribution mechanism leading to a better perceived quality (related to higher prices) when objects are processed primarily in the left hemisphere, and to a lower perceived quality (related to lower prices) when objects are processed primarily in the right hemisphere.

## Introduction

In everyday life people make estimations about different physical attributes of objects with a certain degree of precision. For example, objects have a size defined by three dimensions (length, height, depth) that can be estimated in linear units (meters, feet, and so on), and have a mass that can be estimated in weight units (grams, ounce, and so on). Numerical judgments are easily attached to the subjective estimates of these, as well as many other, object attributes.

Because our society has evolved systems of resources that are grounded on social exchange and monetary transaction of goods, in particular, objects not only possess physical attributes but are also strongly characterized by economic value or price, that can be estimated in a given currency (Euro, Dollar, and so on).

As prices are also expressed numerically, it is reasonable to assume that the quantitative magnitude of price estimation somewhat reflects both the objective (market value, standard price) and the subjective value of a good. In other words, whereas the objective measurement of a physical attribute has a subjective component that usually deviates because of an intrinsic error of the perceptual system, the subjective component of economic value may diverge more dramatically from market value due to a more articulate role of these subjective aspects: we can expect that a bottle of water will be judged as having more or less the same volume or weight when observed in different environmental conditions, but the same observer will be probably willing to pay very different prices for the same bottle in different consumption circumstances (e.g., at home or in the sunny desert) and purchase contexts (e.g., at a supermarket or at the restaurant).

Among the environmental factors, the spatial location of products seems to have an influence on individuals’ perception of price and quality (Cai et al., 2012; Valenzuela and Raghubir, 2015). Specifically, products placed on people’s right side are generally considered more expensive and having higher quality than the same products placed on their left side. Since prices are numerical but they are also assessed in relation to the perceived quality of products (Zeithaml, 1988; Rao and Monroe, 1989), previous studies (Cai et al., 2012; Valenzuela and Raghubir, 2015) suggest that two effects may be responsible for the horizontal asymmetries in price estimation, namely the Spatial Numerical Association of Response Codes (SNARC; Dehaene et al., 1993) and the attribution of valence related to the horizontal space (Casasanto, 2009; Tversky, 2011).

The SNARC effect is a systematic tendency of responding faster and better to smaller numbers with the left hand, compared to the right hand, while the responses to larger numbers are better and faster with the right hand, compared to the left one. The most commonly accepted for this phenomenon, even though still debated, is that numbers may be mentally represented along a continuum, left-to-right oriented, called the Mental Number Line (MNL; Dehaene, 2011). Thus, the estimation of higher prices for products placed on the right (lower prices on the left) might be the result of an implicit and automatic representation of numbers along the horizontal space: smaller on the left and larger on the right.

As regards the attribution of valence in the horizontal space, it can be described as the tendency to associate positive stimuli to the right side of space and negative stimuli to the left side (see for instance Casasanto, 2009; Kong, 2013; Vicario and Rumiati, 2015). This effect has been found in different domains and using different techniques of stimulus lateralization (Bassel and Schiff, 2001; Marzoli and Tommasi, 2009; Prete et al., 2015). Casasanto’s body-specific hypothesis claims that this laterality effect is related to our motor interactions with the environment. Since the actions we perform using our dominant hand are easier and smoother than those performed by our non-dominant hand, right handers tend to attribute positive valence to stimuli placed on their right (i.e., “Good Is right” mapping), while left handers show the opposite tendency (i.e., “Good Is Left”). Thus, for right handers, the attribution of positive valence to products placed on the right may lead to perceive them as having higher quality and higher price, and vice versa for the left side.

However, when people choose a product among different horizontal and vertical positions, the center is usually preferred (Valenzuela and Raghubir, 2009; Rodway et al., 2012), indicating that price estimation side bias does not necessarily imply a side bias.

So far, experimental procedures of price lateralization have been focused on the manipulation of the SNARC effect, for example by using different number arrangements as prime before a price or quality estimation task (see for instance experiment 4 in Valenzuela and Raghubir, 2015 and experiment 4 in Cai et al., 2012), finding out that the numerical processing may be an antecedent of the horizontal bias in price perception.

These pieces of evidence may be just one side of the whole story, although suggestive and insightful. It may well be the case that, while one can safely assume that the basic numerical processing is the same across two different domains of estimation, other mechanisms are contingent upon the specific domains.

For this purpose, we propose a new kind of control, weight estimation, that may bring new insights into the study of prices perception (Cheng and Monroe, 2013).

Weight is a physical attribute which can be represented, just like price, by one single number but, at the same time, its perception also involves other non-numerical cognitive processes. For example, the weight perception seems to be processed by a system which integrates visual cues (object’s features) into an appropriate motor planning necessary for acting in relation to an object (Gallivan et al., 2014). Moreover, unlike price, there is no evidence, to the best of our knowledge, neither of a link between weight estimation and valence attribution nor a clear lateralization of this dimension at a perceptual level (Shen, 1936; Brodie, 1988).

Therefore, if the processing of numerical magnitude were the sole cause of lateral spatial biases for both price and weight, we would expect that it should have an influence on both. In this case, we would expect both attributes to show the same pattern of laterality: higher price/weight estimations when objects are presented in the right visual field (RVF) than left visual field (LVF). Alternatively, if price estimations showed the pattern of lateralization described above and weight did not, then the SNARC effect would no longer be suitable to explain this pattern of laterality. Thus, the alternative explanation is that the former attribute would be influenced by valence attribution, which has spatial properties, whereas the latter would be influenced by a non-lateralized sensory-motor perceptual mechanism.

To test our hypothesis, we use the tachistoscopic lateralized presentation of visual stimuli, a technique which has been widely used to investigate brain symmetries and that, to the best of our knowledge, has never been used in pricing estimation experiments. We will discuss the results considering the possible role of hemispheric asymmetries, which are involved in, and often responsible for, perceptual biases in the horizontal space.

## Materials and Methods

### Participants

Seventy healthy participants (42 females, 28 males) volunteered for the experiment (mean age = 23.31, *SE* = 0.4). Sixty-seven were right-handers (three left-handers were in the Center Group; see section Procedure), their mean hand preference index being 62.30 (*SE* = 3.65) as assessed by the Edinburgh Handedness Inventory (Oldfield, 1971).

Because the task required a price estimation, we recorded other personal data that might have influenced participants’ judgments: *net monthly budget* (mean = € 263.57, *SE* = 19.01). The question asked each participant was “How much money can you count on in one month, excluding the rent for your apartment?”

The whole procedure was carried out in accordance with the principles of the Declaration of Helsinki. The protocol was approved by the Biomedical Research Ethics Committee, University of Chieti-Pescara, and participants gave written and informed consent before beginning the experiment.

### Stimuli

The stimulus material consisted of color pictures taken from the Foodcast Research Image Database (FRIDa) (Foroni et al., 2013) an image database collecting different kinds of food and objects. Our stimuli measured 5.9° × 6.4° of visual angle. Stimuli were 166 images (137 object photos and 29 food photos) selected as follows. First, 204 images from FRIDa (161 representing objects, 43 representing foods) having a value lower than 11.5 in ambiguity were chosen. Ambiguity is an attribute that indicates the image recognition rate, and it was assessed asking the following question: “how easy/difficult is to understand what is represented in the image?” The extremes of the scale were “very easy” (0) and “very difficult” (100). Then a small panel (eight participants) performed an identification task in which stimuli were presented in conditions similar to the main experiment, with the purpose of assessing image recognizability. The task consisted in naming aloud each image. The response was registered using GoldWave (V.5.08, GoldWave, Inc.) software. Stimuli under 62.5% of recognition rate were excluded (mean recognition = 84%). The 166 images were randomly split into two sets of 83 images each, despite this, one set resulted in a higher mean price, and was thus labeled HMP (Higher Mean Price) items set, whereas the other set resulted in a lower mean price, and was thus labeled LMP (Lower Mean Price) items set (see Supplementary Table S1 for the complete lists).

### Procedure

**FIGURE 1:**
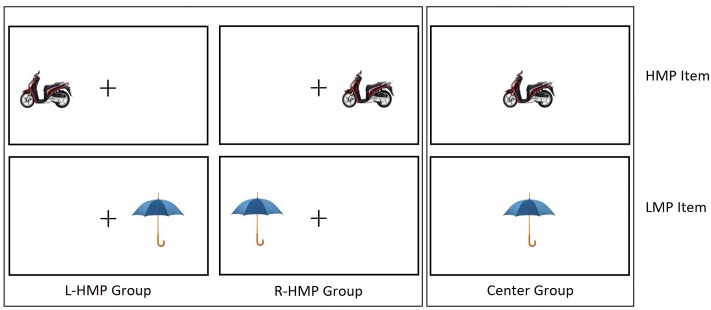
Schematized examples of two stimuli. L-HMP Group were presented HMP items **(Top)** in the LVF and LMP items **(Bottom)** in the RVF. R-HMP Group were presented HMP items in the RVF and LMP items in the LVF. Center Group were presented both HMP items and LMP items Centerly.

The sample was randomly split into three groups: L-HMP Group (Left Higher Mean price; 24 participants) were presented the HMP items in the LVF and the LMP items in the RVF; R-HMP Group (Right Higher Mean Price; 24 participants) were presented the stimuli in the opposite arrangement, i.e., the HMP items in the RVF and the LMP Items in the LVF; Center Group (22 participants) were presented both the HMP and the LMP items in the center of the screen (see **Figure 1**).

Each stimulus was presented on a computer screen with a resolution of 1280 × 768 pixels using E-prime software (Psychology Software Tools, Inc., Pittsburgh, PA, United States). Each stimulus was presented for 150 ms and it was preceded by a fixation cross that lasted 500 ms, then another fixation cross remained on the screen until participants’ response. L-HMP and R-HMP Groups were presented with the stimuli at 6° eccentricity either to the left or the right of a fixation cross (1.6° × 1.6° of visual angle) positioned in the center of the screen. Center Group was presented with the stimuli in the center of the screen (D’Anselmo et al., 2015).

Participants sat comfortably in front of the computer monitor with the head at a distance of approximately 50 cm. The experiment was divided into two separate conditions, i.e., the price condition and the weight condition, performed as two experimental sessions separated by a pause. Each stimulus was presented once for each condition in a pseudorandom order, so that each participant viewed the same list of objects twice: one in the price condition and one in the weight condition. The two conditions were identical except for the required type of estimation (price vs. weight). The presentation order of each condition was counterbalanced across participants. Each session lasted about 10 min.

In the price condition, the task consisted in estimating the price of each item presented. The instruction was: “Try to estimate the exact price of the objects, assuming that all objects are new.” The currency adopted for the estimation was the euro. Participants had to say the price aloud, being free to use currency submultiples also (i.e., eurocents), and then pressed a key to proceed to the next trial. The response was recorded using GoldWave (V.5.08, GoldWave, Inc.) software.

In the weight condition, the procedure was the same as in the price condition but in this case the instruction was: “Try to estimate the exact weight of the objects.” The unit of estimation was the gram and participants were free to use its multiples also (i.e., hectograms, kilos, etc…). We encouraged participants always to fixate the center of the monitor and respond as quickly and accurately as possible after the presentation of each image. When they were not sure whether they had recognized an object, the given response was “not recognized,” and therefore no estimation was provided for that trial. After reading instruction, a familiarization session was performed before starting the experiment.

## Results

To assess for possible differences between groups, we conducted three independent one-way analyses of variance (ANOVAs). The dependent measure was “age” of the sample for the first analysis, “handedness” of the sample for the second one, and “net monthly budget” of the sample for the third one. For all of them, the independent variable was the group and it had three levels: L-HMP, R-HMP, Center. Results showed no significant differences between groups (**Table 1** reports descriptive data for all groups and variables).

**Table 1:** Descriptive statistics of all participants’ characteristics for each individual group (mean values are reported; standard errors are in brackets).

		Participants’ characteristics		
		
		L-HMP Group	R-HMP Group	Center Group
Number of				
participants		24	24	22
Sex	F	15	14	13
	M	9	10	9
Age		23.85 (0.73)	23.54 (0.79)	22.45 (0.52)
Handedness		68.98 (3.32)	67.32 (3.33)	49.55 (10.07)
Net monthly budget		256.25 (38.91)	273.75 (35.90)	260.45 (20.49)


All prices were converted to euro and all weights to hectograms. 5% Winsorized means were used for each estimated object.

For the main by-subject analysis, price and weight were treated separately, and mean estimations of each Item Set (HMP items, LMP items) were computed for each participant and estimation type.

Participants having total estimation means ± 2 SD from the whole sample mean were considered outliers and they were computed independently for each estimation type. Supplementary Figures S1, S2 show mean and SE of each participant’s overall estimations for price and weight respectively.

Thus, participants excluded in one estimation type are different from those excluded in the other one. Four participants were excluded for price analysis: two in L-HMP Group and two in R-LMP Group. Four participants were excluded for weight analysis: one in L-HMP Group, two in R-HMP Group and one in Center Group.

Two mixed-design ANOVAs were also carried out in order to assess a possible effect of estimations’ order (price first or weight first). For both price and weight: Item Set (HMP, LMP) as within-subject factor, Group (L-HMP Group, R-HMP Group, Center Group) as first between-subjects factor, and Estimation as second between-subject factor (Price first, Weight first); Item Set (HMP, LMP) as within-subject factor, Group (L-HMP Group, R-HMP Group, Center Group) as first between-subjects factor, and Sex as second between-subject factor (Male, Female).

The first analysis, with Estimation as second between factor showed no significant main effect and no significant interactions with either Item Set or Group, in both price and weight estimations. The second analysis, with Sex as second between-subject factor showed no significant main effect and no significant interactions with neither Item Set nor Group, in both price and weight estimations. As these factors did not influence hemispheric asymmetries, they were therefore not included in the subsequent analyses.

For the main analysis, data were then analyzed by using two (price and weight) mixed-design ANOVAs with Item Set (HMP, LMP) as within-subject factor and Group (L-HMP Group, R-HMP Group, Center Group) as between-subjects factor. **Table 2** reports descriptive data for the two conditions for the three groups.

**Table 2:** Descriptive results.

	L-HMP Group		R-HMP Group		Center Group	
			
Price	LVF	RVF	LVF	RVF	Center	Center
Items	HMP	LMP	LMP	HMP	HMP	LMP
Mean price estimation in euro	63.14 (4.47)	36.66 (2.46)	36.52 (2.81)	76.73 (5.42)	77.90 (6.83)	32.51 (2.65)
**Weight**					
Items	HMP	LMP	LMP	HMP	HMP	LMP
Mean weight estimation in hectograms	51.30 (8.07)	14.03 (1.52)	15.60 (2.39)	41.34 (10.15)	38.74 (5.55)	14.64 (1.36)


*Mean estimations in the two experimental conditions. Estimations are in Euro for Price (above) and in Hectograms for Weight (below); standard errors are in brackets. In both conditions, L-HMP Group viewed HMP items in the LVF and LMP items in the RVF. R-HMP Group viewed the LMP items to the LVF and HMP items to the RVF. Center Group viewed both HMP and LMP items in the Center.*

### Price Estimation

**FIGURE 2:**
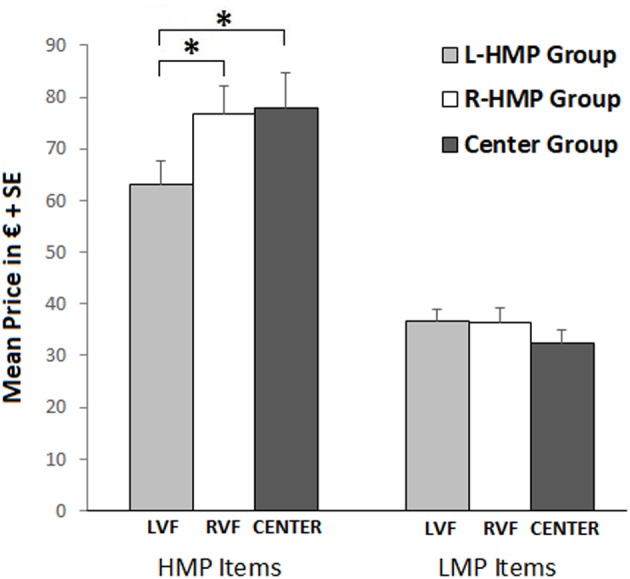
Mean price estimation in L-HMP Group (gray bars), R-HMP Group (white bars), and Center Group (dark gray bars). Figure refers to the significant interaction Item Set × Group. Left bars refer to HMP item set, right bars refer to LMP item set. Asterisks refer to significant *post hoc* comparisons.

Analysis of variance showed no significant main effect of Group and a significant main effect of Item Set (*F*_1,63_ = 256.15; *p* < 0.001; $\eta_{p}^{2}$ = 0.78), with higher prices estimated for HMP items compared to LMP items. Moreover, a significant interaction effect was found between Item Set and Group (*F*_2,63_ = 5.84; *p* = 0.005; $\eta_{p}^{2}$ = 0.15; see **Figure 2**). Duncan’s *post hoc* comparisons (asterisks in **Figure 2**) showed differences between groups regarding the price of HMP items. Specifically, L-HMP Group judged HMP items as less expensive than R-HMP Group (*p* = 0.032) and Center (*p* = 0.02). No significant differences were found between groups R-HMP and Center. In terms of laterality, HMP items viewed in LVF (L-HMP Group) were underestimated compared to the RVF (R-HMP Group) and Center (Center Group).

### Weight Estimation

**FIGURE 3:**
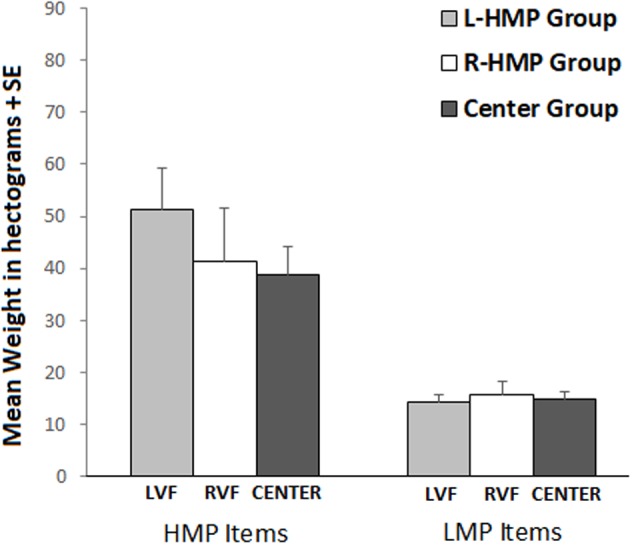
Mean weight estimation in L-HMP Group (gray bars), R-HMP Group (white bars), Center Group (dark gray bars). Figure refers to the non-significant interaction Item Set × Group. Left bars refer to HMP item set, right bars refer to LMP item set.

Analysis of variance showed no significant main effect of Group and a significant main effect of Item Set (*F*_1,62_ = 46.02; *p* < 0.001; $\eta_{p}^{2}$ = 0.37), with higher weights estimated for HMP items compared to LMP items. No other significant effect was found (see **Figure 3**).

### By Item ANALYSIS

An item-by-item analysis was carried out in order to compare price and weigh laterality and specify the nature of the interaction between item sets and side of objects’ presentation. This analysis concerns only L-HMP and R-HMP groups. All participants were included.

For each object and estimation type, we calculated its mean, mediating left and right estimations, and its Laterality Index using the formula LI = (R - L)/(R + L) × 100, where R is RVF and L is LVF (D’Anselmo et al., 2016). This formula allows us to compare left and right absolute differences between the two types of estimation.

The first analysis concerns the main laterality difference between price and weight.

For price estimations, we excluded objects having Laterality Indices greater than ±2 SD from the sample mean (11 objects). We applied the same procedure for weight, excluding 13 objects. Then, we matched each object’s price LI with its weight LI, obtaining a matched sample of 144 objects, in order to compare the main laterality difference between the two types of estimations. Price LI mean = 1.03 (*SE* = 1.06) vs. weight LI mean = -4.59 (*SE* = 2.13) were compared by a paired *t*-test (t143–2.41; *p* = 0.02), indicating a significant difference in lateralization between the two types of estimation.

The second analysis was carried out to test whether the differences in lateralization between Item sets in price estimation are related to their differences in mean price.

**FIGURE 4:**
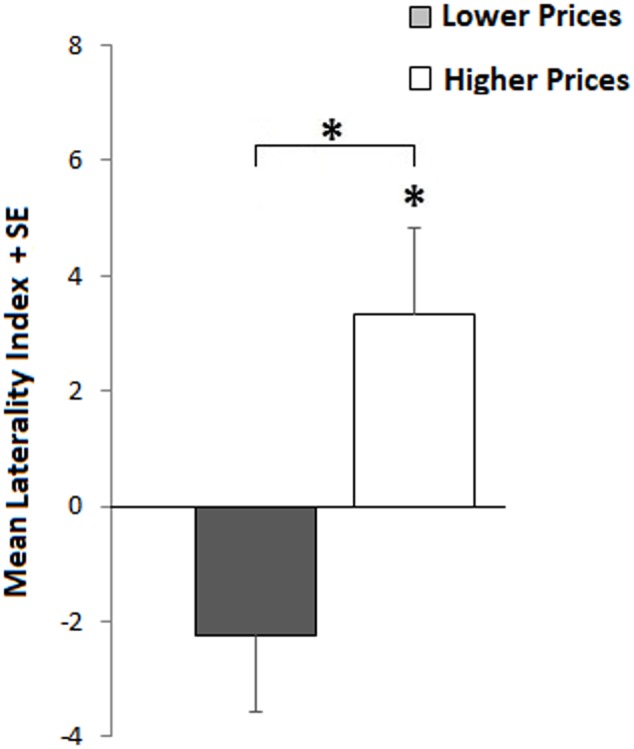
Item-by-item. Laterality differences between lower (gray bar) and higher (white bar) sets of objects’ prices. Positive indices indicate RVF advantage (higher estimations); negative indexes indicate LVF advantage. The asterisks refer to the significant difference between groups (upper asterisk) and against 0 (lower asterisk).

We started from the sample of 155 objects filtered for LI outliers (described above) and split it along its median price previously computed, obtaining two groups of objects having different prices: lower and higher. Thus, we compared the LI of these two groups (lower = -2.24 vs. higher = 3.35) by a two-sample *t*-test (t153–2.81; *p* = 0.006), indicating a right advantage for higher estimations and a left advantage for lower estimations. However, only the right advantage resulted significantly greater than 0 (3.35 ≠ 0; t77–2.28; *p* = 0.03), indicating that more expensive objects are estimated as more expensive in the RVF, whereas cheaper objects are estimated equally expensive in both LVF and RVF (see **Figure 4**). Descriptive statistics are reported in **Table 3**.

**Table 3:** By item analysis.

	Lower prices	Higher prices
Real prices	4.25 (0.36)	115.46 (19.47)
Minimum	0.5	12.34
Maximum	12.33	1255
Laterality Index	-2.24 (1.34)	3.35 (1.47)
LVF estimations	5.21 (1.07)	96.89 (14.80)
RVF estimations	5.17 (1.23)	110.32 (20.04)


*Descriptive statistics of lower and higher sets of objects’ prices (mean values are reported; standard errors are in brackets).*

Then we checked for the relation between price and LI as continuous variables, converting mean prices in percentile rank. The positive correlation (*r* = 0.22; *p* = 0.007) indicates that LIs increase, from negative (LVF) to positive (RVF) indices, along with mean prices of objects (see Supplementary Figure S3).

## Discussion

Our results partially corroborate previous findings (Cai et al., 2012; Valenzuela and Raghubir, 2015) about the existence of an asymmetry in price estimation: objects shown in the LVF tend to be perceived as less expensive than those shown in the RVF, a result that may be due to the SNARC effect. Nevertheless, the lack of asymmetry in weight condition suggests that this explanation cannot be completely exhaustive, at least in our specific task, and that the attribution of valence can be a convincing alternative explanation.

However, the effect of laterality in price estimations was found only for the set of objects that was estimated as more expensive (set HMP) than the other one (set LHP), for which no laterality was found. This unexpected result might be due to a large sample of objects that was randomly split in two groups. Specifically, it seems that only products having higher prices are, overall, estimated in line with our alternative hypothesis.

In fact, *post hoc* analyses reveal a positive correlation between prices and Laterality Indices indicating that an increase in prices corresponds to an increase of the RVF advantage, which is significantly greater than the LVF advantage only for more expensive objects.

Nevertheless, these objects ranged from 12 to 1255 €, whereas the other group (less expensive) ranged from 0.50 to 11.70 €, indicating that the laterality effect occurs, overall for a rather wide set of price estimations.

In light of these facts, the difference between the two sets of objects can be explained by the variability of estimations that tend to become more approximate and imprecise as the magnitude increases, a property of numerical estimations, that derives from the Weber’s law (Dehaene and Marques, 2002; Castronovo and Seron, 2007). It is possible that the appraisal of lower prices, being less variable, leaves less room for a valence based estimation bias, holding estimates closer to actual prices. On the contrary, higher prices might facilitate inferences based on valence attribution, that in turn lead to a side bias. However, the nature of this variability, as explained by Dehaene in his study of price estimation (2002), can be partly numerical and partly explained by purchasing factors. Higher price products also have more variability in the marketplace and less frequency of purchasing, making them more difficult to estimate on the base of an objective criterion.

Thus, it is possible that, in a free estimation price task, a greater objective uncertainty corresponds to a greater influence of environmental cues, which, in this case, is the side where the product is presented.

As we reasoned in the introduction, the source of this bias is twofold. On one hand, there is the influence of the mental horizontal mapping of numbers, on the other hand the effect of valence attribution which has spatial properties as well, and the lack of effect in weight estimation suggests that these effects might be dissociable. The attribution of valence can account for the result found in price estimation, because of the association *positive valence = higher quality = higher price* for right-placed products, and *negative valence = lower quality = lower price* for left-placed products. Since prices are somehow indicators of “good” and “bad” products, we might say that they can influence the perceived quality, and vice versa. In this regard, a study using functional magnetic resonance (fMRI; Plassmann et al., 2008) showed that increasing the price of a bottle of wine, the perception of its flavor and pleasantness also increases, activating the orbitofrontal cortex (mOFC), a region that is more generally involved in experiences of pleasantness.

Casasanto’s (2009) body-specific hypothesis suggests that the horizontal space is naturally associated to the idea of “good” and “bad” depending on our dominant hand. Right handers experience better and more fulfilling interactions with the environment using their right dominant hand than the non-dominant one. Consequently, they may have embodied the idea that “Good is right” and “Bad is left,” while the left handers may have embodied the opposite association. For instance, Kong (2013) found that right handers respond faster to face and words having positive (negative) valence using their right hand (left), while left handers show the opposite pattern (positive-left, negative-right). Other studies indicate that positive and negative trading verbs (Vicario and Rumiati, 2015) are also mapped in according to the right-positive/left-negative body asymmetry, and in an interpersonal choice task, right handers prefer to choose faces showed on the right, while left handers prefer those on the left (Zhao et al., 2016).

One interpretation for the lack of laterality in the weight condition might be that the SNARC effect was not sufficient to drive a horizontal bias in our task. However, as stated in the Introduction, the perception of weight does not seem to be influenced by perceptual and brain asymmetries (Brodie, 1988). Therefore, had the effect of numerical magnitude been involved, it should have easily biased the weight estimations, which do not seem to have other sources of asymmetry.

Along this line, it is worth noting that the free estimation task used in the present experiment does not provide a numerical range, which is a crucial factor in determining the direction of the SNARC effect. In his seminal paper, Dehaene et al. (1993), demonstrated that when numbers range from 0 to 5, the digits 4 and 5 are coded as “larger,” facilitating the responses given with the right hand. On the contrary, when the range was from 4 to 9, the digits 4 and 5 were coded as “smaller,” facilitating the responses with the left hand. This clearly illustrates that the SNARC effect is influenced by the relative magnitude used for the task, not by the absolute magnitude of numbers.

Thus, providing no range for estimation, we probably impeded the SNARC from occurring, in both price and weight estimations. This reasoning leaves one possible argument to explain the lateralization found for prices, namely the attribution of valence driven by hemispheric asymmetries.

From a neurocognitive perspective, functional cerebral asymmetries in valence attribution, suggest a different left-hemisphere (LH)/right-hemisphere (RH) specialization for positive and negative emotions respectively (see for instance Wedding and Stalans, 1985; Davidson et al., 1990; Bassel and Schiff, 2001; Davidson, 2004; Najt et al., 2013). Thus, our results, along with previews empirical findings, indicate that hemispheric asymmetries may play a role in price evaluation, influencing the perception of products’ quality, and thus, leading to lower vs. higher price estimation when objects are presented in the LVF (RH) vs. RVF (LH) respectively.

Our results also show that prices are underestimated when objects are presented in the LVF, compared to both RVF and Center which do not differ from each other. This pattern of lateralization may suggest that the LH (RVF) is more accurate in price estimation whereas the RH (LVF) tends to underestimate prices. Interestingly, a similar pattern was found in the study by Valenzuela and Raghubir (2015), in which the difference between center and leftmost positions, both in quality and prices, was more accentuated than that between center and rightmost positions, even though they used a Likert scale instead of actual prices. So that an alternative explanation might be that the RH (LVF), driving a negative valence attribution, leads to an actual underestimation of both quality and price. On the contrary, the positive evaluation of LH (RVF) does not lead to an actual overestimation, compared to the Center unbiased condition of estimation. This explanation is congruent with the idea that a positive evaluation of quality can lead to higher price estimation to a certain extent without exceeding the actual price (Ravaja et al., 2013).

However, as we stated in the introduction, it must be noticed that when the task requires to choose a product among several alternatives aligned horizontally, there is a tendency to choose the one in the middle. This is known as the Center-stage effect (Valenzuela and Raghubir, 2009). Casasanto’s theory predicts that the better valence attributed to the right would lead right handers to prefer the products placed on their dominant side, coded as “positive”. However, when a central option is provided, this model fails to predict the actual choice, which is instead accounted for by the Center-stage effect (Rodway et al., 2012).

Thus, the influence of the valence attribution in price estimations should be interpreted more as a mechanism of unconscious inference, which leads to estimate a price based on the perceived quality of a product, than an actual bias of choice. After all, a product having high price and quality is not always a good deal for a mean consumer. In fact, Valenzuela and Raghubir (2009) argue that products having a reasonable balance between price and quality are usually placed in the middle, and consumers seem to be aware of that.

In conclusion, our work disentangles between the contributions of two possible cognitive mechanisms underlying the estimation of prices: the SNARC effect and the attribution of valence. Our results support the attribution of valence, that leads prices to be estimated higher in the RVF than in the LVF, indicating a differential involvement of the two cerebral hemispheres. Specifically, we speculate that the left hemisphere, specialized for positive emotions, leads to a better perceived quality, and thus to higher estimations. The right hemisphere, specialized for negative emotions, leads to a worse perceived quality, and thus to lower estimations.

This study provides support for the idea that the perception of economic value is based on embodied experiences of the world.

## Author Contributions

FG: substantial contributions to the conception or design of the work; critical revision for important intellectual content of the work; acquisition, analysis and interpretation of data for the work; drafted the work. AD, LT, AB, and DP: substantial contributions to the conception or design of the work; critical revision for important intellectual content of the work; agreement to be accountable for all aspects of the work in ensuring that questions related to the accuracy or integrity of any part of the work are appropriately investigated and resolved; final approval of the version to be published.

## Conflict of Interest Statement

The authors declare that the research was conducted in the absence of any commercial or financial relationships that could be construed as a potential conflict of interest.
