# Supplementary material for: Hemispheric Asymmetries in Price Estimation: Do Brain Hemispheres Attribute Different Monetary Values?
Source: Front Psychol. 2017 Nov 22;8:2042. doi: 10.3389/fpsyg.2017.02042 (PMC5702889; doi:10.3389/fpsyg.2017.02042)
Supplement: Supplementary file 1 [file Table_1.docx]

Supplementary Material

**Hemispheric asymmetries in price estimation: do brain hemispheres attribute different monetary values?**

**Felice Giuliani*, Anita D’Anselmo, Luca Tommasi, Alfredo Brancucci & Davide Pietroni**

*** Felice Giuliani:** felice.giuliani@unich.it

# Supplementary Table 1

List of stimuli. Code name indicates the category (AFO = Artificial Food Objects; AO = Artificial Objects; NF = Natural Food; TF = Transformed Food).

|  |  |  |  |  |  |  |
| --- | --- | --- | --- | --- | --- | --- |
|  | **HMP Item** | |  | **LMP Item** | |  |
|  | **Code** | **Name** |  | **Code** | **Name** |  |
|  | AFO_024 | coffee maker |  | AFO_015 | scale-02 |  |
|  | AFO_037 | knife-03 |  | AFO_018 | kettle-02 |  |
|  | AFO_054 | blender-03 |  | AFO_023 | jug |  |
|  | AFO_057 | grater-02 |  | AFO_061 | oven mitt |  |
|  | AFO_065 | rolling pin |  | AFO_115 | toaster |  |
|  | AFO_074 | frying pan |  | AO_001 | lighter |  |
|  | AFO_078 | pot-01 |  | AO_002 | toy airplane |  |
|  | AO_006 | watering can |  | AO_004 | speakers |  |
|  | AO_010 | wardrobe |  | AO_007 | satellite dish |  |
|  | AO_012 | hair dryer |  | AO_014 | vacuum cleaner |  |
|  | AO_018 | car radio |  | AO_019 | porcelain doll |  |
|  | AO_023 | drum set |  | AO_027 | bicycle |  |
|  | AO_028 | foosball table |  | AO_029 | binocular |  |
|  | AO_031 | purse |  | AO_030 | bowling pin |  |
|  | AO_037 | screwdriver |  | AO_038 | calculator |  |
|  | AO_039 | short socks |  | AO_041 | shirt-02 |  |
|  | AO_053 | construction helmet |  | AO_044 | candelabra |  |
|  | AO_057 | cassette |  | AO_047 | tank top |  |
|  | AO_078 | picture frame |  | AO_048 | top hat |  |
|  | AO_085 | cradle |  | AO_060 | cell phone |  |
|  | AO_086 | pillow |  | AO_065 | ratchet wrench |  |
|  | AO_099 | iron |  | AO_067 | key-01 |  |
|  | AO_106 | nail scissors |  | AO_070 | guitar |  |
|  | AO_109 | dart |  | AO_077 | rope |  |
|  | AO_112 | toilet |  | AO_092 | helmet |  |
|  | AO_114 | parka |  | AO_093 | bicycle helmet |  |
|  | AO_115 | jacket |  | AO_094 | fire extinguisher |  |
|  | AO_127 | hanger |  | AO_101 | thread |  |
|  | AO_136 | chandelier |  | AO_102 | whistle |  |
|  | AO_137 | lightbulb |  | AO_105 | scissors |  |
|  | AO_139 | washing machine |  | AO_111 | birdcage |  |
|  | AO_144 | mp3 player |  | AO_117 | broom |  |
|  | AO_145 | bookcase |  | AO_118 | bed slats |  |
|  | AO_149 | sewing machine |  | AO_119 | eraser |  |
|  | AO_150 | camera |  | AO_129 | gloves |  |
|  | AO_152 | sweater |  | AO_131 | boxing gloves |  |
|  | AO_157 | pencil |  | AO_140 | binder |  |
|  | AO_165 | chainsaw |  | AO_146 | book |  |
|  | AO_167 | sunglasses |  | AO_147 | life preserver |  |
|  | AO_168 | eyeglasses |  | AO_154 | hammer |  |
|  | AO_176 | dustpan |  | AO_156 | mattress |  |
|  | AO_177 | soccer ball |  | AO_159 | mallet |  |
|  | AO_180 | golf ball |  | AO_164 | clothes pin |  |
|  | AO_191 | pen |  | AO_170 | umbrella |  |
|  | AO_203 | pipe |  | AO_173 | wristwatch |  |
|  | AO_204 | gun |  | AO_178 | tennis ball |  |
|  | AO_210 | coin purse |  | AO_179 | basketball |  |
|  | AO_217 | notebook |  | AO_182 | shorts |  |
|  | AO_218 | tennis racket |  | AO_184 | stroller |  |
|  | AO_223 | rake |  | AO_200 | pinwheel |  |
|  | AO_227 | lipstick |  | AO_206 | polo shirt |  |
|  | AO_233 | chessboard |  | AO_208 | laundry basket |  |
|  | AO_234 | rack |  | AO_219 | hair straightener |  |
|  | AO_241 | scooter |  | AO_221 | shaver |  |
|  | AO_243 | bucket |  | AO_239 | scarf |  |
|  | AO_244 | folding chair |  | AO_245 | saw |  |
|  | AO_255 | showerhead |  | AO_249 | stool |  |
|  | AO_259 | mirror |  | AO_251 | skateboard |  |
|  | AO_267 | alarm clock |  | AO_256 | sword |  |
|  | AO_277 | remote control |  | AO_258 | toothbrush |  |
|  | AO_279 | telescope |  | AO_261 | sponge |  |
|  | AO_280 | pencil sharpener |  | AO_266 | rain boots |  |
|  | AO_282 | curtains |  | AO_272 | keyboard |  |
|  | AO_286 | flashlight |  | AO_274 | occasional table |  |
|  | AO_291 | suitcase |  | AO_276 | palette |  |
|  | AO_294 | vase |  | AO_278 | telephone |  |
|  | NF_004 | pineapple |  | AO_281 | tent |  |
|  | NF_013 | banana |  | AO_287 | lawn mower |  |
|  | NF_022 | cauliflower |  | AO_288 | drill |  |
|  | NF_049 | lemon-01 |  | AO_293 | pot |  |
|  | NF_055 | apple-01 |  | AO_295 | fan |  |
|  | NF_056 | apple-02 |  | NF_007 | lobster |  |
|  | NF_073 | chilli pepper-01 |  | NF_010 | asparagus |  |
|  | NF_077 | pear |  | NF_019 | carrot |  |
|  | NF_079 | peach |  | NF_027 | cherry |  |
|  | NF_095 | egg |  | NF_037 | strawberry |  |
|  | TF_033 | chocolate bar-01 |  | NF_059 | eggplant |  |
|  | TF_036 | cornet-01 |  | NF_076 | pepper-02 |  |
|  | TF_069 | hamburger-02 |  | NF_084 | tomato |  |
|  | TF_070 | hotdog |  | TF_006 | baguette |  |
|  | TF_109 | roasted chicken |  | TF_050 | cupcake |  |
|  | TF_127 | salami |  | TF_063 | ice cream-02 |  |
|  | TF_131 | sausage |  | TF_092 | fruit pie |  |
|  |  |  |  |  |  |  |
