# Supplementary material for: Hemispheric Asymmetries in Price Estimation: Do Brain Hemispheres Attribute Different Monetary Values?
Source: Front Psychol. 2017 Nov 22;8:2042. doi: 10.3389/fpsyg.2017.02042 (PMC5702889; doi:10.3389/fpsyg.2017.02042)
Supplement: Supplementary file 3 [file Image_2.PDF]

Supplementary Material

Hemispheric asymmetries in price estimation: do brain hemispheres attribute different monetary values?

Felice Giuliani*, Anita D'Anselmo, Luca Tommasi, Alfredo Brancucci & Davide Pietroni

*** Felice Giuliani:** felice.giuliani@unich.it

Supplementary Figures

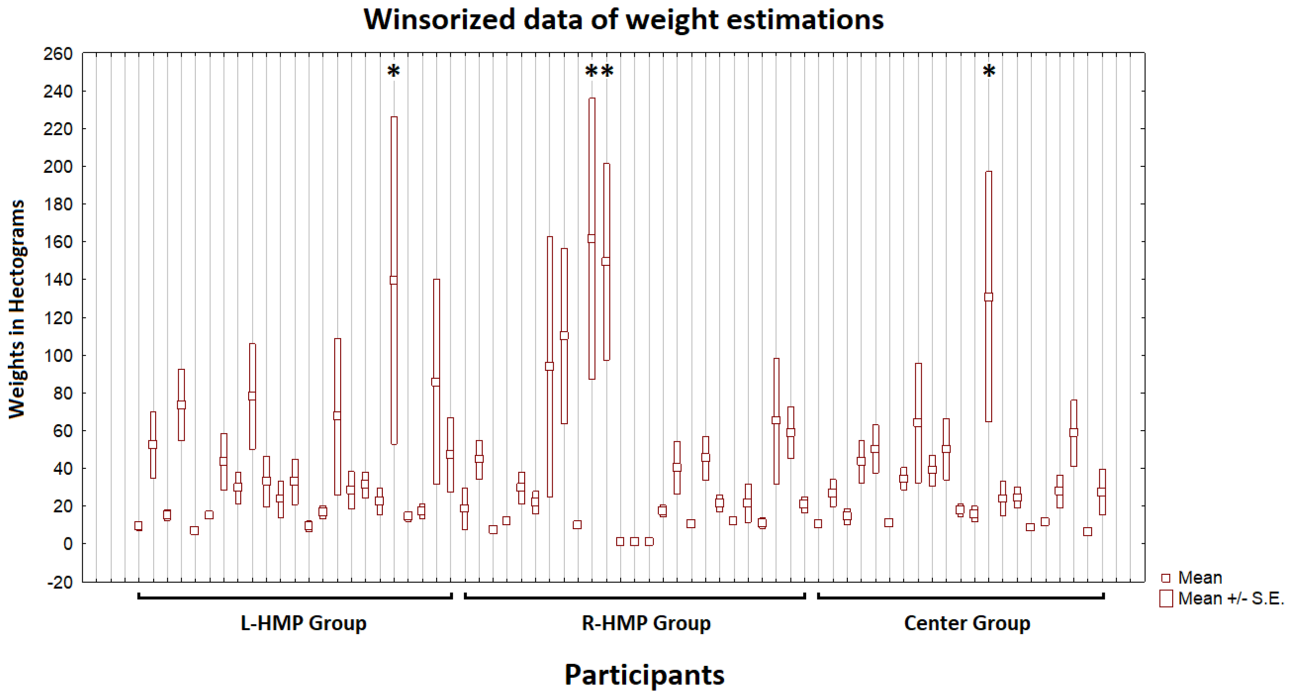

Supplementary figure S2. Mean weight estimations for each participant, relative to all objects' sample. Asterisks indicate outlier participants (+/- 2 SD from the sample mean).
